# Immunotoxicity Following Pre- and Post-natal Aluminum Exposure in Rats

**DOI:** 10.5487/TR.2008.24.1.051

**Published:** 2008-03-01

**Authors:** Abd El-Azeim A. Khalaf, Ashraf M. Morgan, Mohey M. Mekawy, Maged F. Ali

**Affiliations:** Department of Toxicology and Forensic Medicine, Faculty of Veterinary Medicine, Cairo University, P.O. Box 12211, Giza, Egypt

**Keywords:** Aluminum chloride, Immunotoxicity, Rats, Humoral and cell mediated immune response, Thymus, Spleen, Liver

## Abstract

The present study was designed to explore the immunotoxic effects of orally administered aluminum (AI) on pregnant rats (n = 60) and their growing fetuses and consequently on the animal wealth. The animals were randomly allocated into three equal groups of 20 rats each. The first group has no treatment and kept as a control (G1). The second and third groups of pregnant rats were treated orally with aluminum chloride at 345 mg/Kg b.wt. The second group (G2) received the tested compound from the 6^th^ day of gestation to the end of weaning, whereas the third group (G3) received the tested compound from the 15^th^ day of gestation to the end of weaning. Control and treated animals (dams and offspring) were immunized ip with (0.5 ml) 20% sheep red blood cell (SRBC) suspension seven days before the end of experiments. At the end of exposure, ten dams and ten offspring from each group were used for assessment of cell-mediated immunity and a similar number of animals were sacrificed for evaluating the humoral immune response and serum protein profile. Aluminum chloride exposure of dams (G_2_ & G_3_) caused significant suppression of both cell mediated and humoral immune responses in the obtained offsprings compared to the control group (G_1_) without any significant effect on the immune responses of these dams. Moreover, the serum total globulins, albumin/ globulin (A/G) ratio and gamma globulin fraction were significantly decreased in the treated dam’s offsprings compared to the corresponding controls while the serum total protein and all serum protein fractions showed non significant difference between the control and treated dams and between the two treated dam groups themselves. There were no histopatho-logical changes observed in thymus, spleen and liver of the control and treated dams. Thymus of treated dam’s offsprings (G2) showed lymphoid depletion in both cortex and medulla. Their spleens showed lymphoid depletion in the white pulps and congestion with hemosiderosis in the red pulps. Liver of treated dam’s offsprings showed dilation and congestion of its central vein with degenerative changes in the hepatocytes. These histopathological changes were more severe in G2 than in G3 offsprings. It can be concluded that gestational and/ or lactation exposure of pregnant dams to AI chloride caused suppression of both cellular and humoral immune responses of their offsprings.

## INTRODUCTION

Metals are the toxic chemical compounds which are deliberately introduced into the environment where they can cause toxicity and death of non target organisms (Domingo, 1995; Schafer *et al*., 1999; Carson, 2000). The flood of metals during the past decades due to their extensive use in industries was correlated with the pollution of food stuffs and the induction of hazardous effects on humans and animals (Ganrot, 1986; WHO, 1997). Their extensive use poses a serious threat not only to those who are in immediate contact but to every living organism through contaminated food, water, and air (Schafer *et al*., 1999). Aluminum (Al) compounds include Al sulphate, chloride, hydroxide, nitrate, and others. The main hazard to livestock from these compounds likely to arise from their use in treatment of drinking water, drugs (*e.g.*, antacids), deodorants and antiperspirants preparations, preservation of wood, the disinfection of stables and slaughterhouses, and in manufacture of alloys (Lione, 1985; ATSDR, 2006). The danger from use of such compounds, not only because most of them are extremely poisonous, but also because, with many, the effects of exposure are not immediately apparent, but are insidious in onset (Misawa and Shigeta, 1992; Zaman *et al.*, 1993; Carson, 2000).

Aluminum adjuvants are the only adjuvants allowed for use in human vaccines and is present in many veterinary vaccines (Bomford, 1980). These adjuvants augment the type II immune response without enhancing the type 1 immune response (Bomford, 1980; Comoy *et al.*, 1997). The use of triple vaccines for childhood immunization may induce sensitization to the aluminum hydroxide added to the vaccine (May *et al.*, 1986). In addition, contact dermatitis was observed to be aggravated by systemic aluminum from toothpaste. Barr *et al.* (1993) supported the concept that aluminum chloride (Drysol) can cause a proliferative histiocytic reaction when used as a topical cauterizing agent. It has been observed that Al exposure of rats in drinking water caused hyperemia in the red pulp of the spleen (Gomez *et al.*, 1986). Nordal *et al.* (1989) and Msarcpondes *et al.* (1996) attributed the observed rejection episode in kidney allograft recipients to Al accumulation in bone, suggesting that Al accumulation has an immunosuppressive effect. Moreover, Golub *et al.* (1993) and Tsundoa and Sharma (1999) recorded that aluminum exposure of experimental animals during gestation and lactation periods affected cytokines production in the offspring (Golub *et al.*, 1993; Tsundoa and Sharma, 1999). It decreased spleen concentrations of interleukin-2, interferon-gamma and tumor necrosis factor-alpha with deficiency of CD4+ cells in T cell populations in the offspring (Golub *et al.*, 1993). Similarly, SC treatment of mice with alum (Al sulphate) induced IL-4 production and T-helper cell type 2 (Th2) responses in the absence of IL-4 in mice deficient in IL-4R $\alpha$ (Carson, 2000). Synzynys *et al.* (2004) confirmed the immunosuppressive effect of aluminum chloride specially on primary T-dependent humoral immune response with diminished thymic and splenic cellularity when given to mice at the genotoxic dose (0.04 M).

There was little concern about the pre- and postnatal immunotoxic consequences of aluminum ingestion because its bioavailability was considered low. Therefore, the present study was designed to highlight on the immunotoxic effects of the pre- and postnatal orally administered Al chloride on the dams and their growing fetuses and consequently on the animal health.

## MATERIALS AND METHODS

***Chemicals.*** The tested aluminum salt is aluminum chloride ($AlCl_3 \cdot 6H_2O$). Aluminum chloride was purchased from Sigma Chemicals Co. (St. Louis, USA) with a M.wt. of 241.43. It was given orally to the treated rats at a dose of 345 mg/Kg b.wt (Sharma and Mishra, 2006).

***Animals.*** Adult male and female albino rats, having body weight 180~200 g, were used as Lab. animals in this study. They were obtained from Faculty of Veterinary Medicine, Cairo University. The animals were kept under hygienic conditions and provided with balanced ration and water *ad libitum*. All animals were kept under observation along two weeks before the start of the experiment for acclimatization.

***Animal exposure and experimental protocol.*** The effect of Al on the immune function of exposed animals was assessed by running two experiments for evaluation of both humoral and cell-mediated immune responses. The humoral immune response was assessed through application of the hemolysin assay; which is a modified micro-technique of complement fixation test; designed for determination of the titer of hemolysin antibodies in serum. At the mean time, the technique of Delayed-type Hypersensitivity (DTH) to sheep red blood cells (SRBC) was concomitantly carried out for investigation of the effect of Al on the cell mediated immune response, as proposed by Seinen *et al.* (1977).

Sixty pregnant rats and their offsprings were used for immunological assays. Pregnant rats were randomly classified into three equal groups, 20 rats each. The first group received no treatment and served as control group (G1). The second and third groups of pregnant rats were treated orally with aluminum chloride (at dose level of 345 mg/Kg b.wt). The second group (G2) received the tested compound from the $6^{th}$ day of gestation to the end of weaning while the third group (G3) received the tested compound from the $15^{th}$ day of gestation to the end of weaning (G3).

Sheep red blood cells (SRBC) were obtained from healthy sheep through puncture of jugular veins and collection of whole blood. Heparin was used as an anticoagulant at a concentration of 1%. Blood was centrifuged at 3000 rpm for 15 minutes. The supernatant layers were decanted and erythrocytes were washed three times with physiological saline and re-centrifuged then finally preserved in Alsever's solution in the form of 20% suspension. The SRBC suspension was used in immunization of all animals in the treated and control groups. In the hemolysin micro titration, 1% SRBC suspension in complement fixation diluent was used.

Control and treated animals (dams and offspring) were immunized ip with 0.5 ml 20% SRBC suspension seven days before termination of experiment. At the end of exposure, ten dams and ten offspring from each group were used for assessment of cell-mediated immunity and a similar number of animals were sacrificed for studying the humoral immune response (Monis and Valentich, 1993). Thymus, spleen and liver of both dams and fetuses were taken for histopathological examination.

***Assay of cell-mediated immune response.*** Animals subjected to cell-mediated immune response study were injected intradermally with 0.5 ml of SRBC suspension in the hind foot pad. The delayed type hypersensitivity (DTH) reaction, a measure of cell-mediated immunity, was evaluated by measuring the diameter of skin reaction 24-hours after inoculation (Dean *et al.*, 1989).

***Assay of humoral immune response (serum hemolysin antibody titer).*** Serial twofold dilution (100 μl of decomplemented sera; 56°C for 30 minutes) were prepared in micro titration wells. 50 μl of 1% SRBC suspension and 50 μl of guinea pig complement diluted 1 : 10 were added. The hemolysin titers were read after incubation for one hour at 37°C. For statistical analysis of the read antibody titers to be rational, each titer should be arithmetically transformed to log 2 values, using the following equation:

$$\text{Log 2 antibody titer in serum} = \frac{\text{Log No. of dilution reciprocal}}{\text{Log2}}$$

Titers represent the reciprocal of the highest dilution giving total hemolysis (Seinen *et al.*, 1977).

***Assay of total proteins.*** It was carried out by a test kit according to Biuret method described by King and Wooton (1959).

***Serum protein electrophoresis.*** It was performed according to the method described by Keyser and Watkins (1972).

***Histopathological investigation.*** Thymus, spleen and liver were fixed in 10% neutral formalin and subjected to histopathological examination according to Bancroft *et al.* (1996).

***Statistical analysis.*** The significant differences between treated and control groups were assessed by One-Way-Analysis Of Variance (ANOVA) using Computer Microstat Program, Copyright (C) 1978-85 by Ecosft, Inc.

## RESULTS

***Effects on cell mediated immune response (DTH reaction).*** Exposure of dams to aluminum chloride ($G_2$ & $G_3$) at the previously mentioned dose level caused non significant suppression of the cell mediated immune response (DTH reaction) compared to the control group ($G_1$). On the other hand, aluminum chloride exposure of these dams caused significant inhibition of the cell mediated immune response in their offsprings ($G_2$ & $G_3$) in relation to the offsprings from the control group ($G_1$) as shown in Table 1.

***Effects on the humoral immune response (serum hemolysin antibody titer).*** Table 1 shows the hemolysin antibody titer in control and treated dams and their offsprings. Exposure of dams to aluminum chloride at the same dose level (345 mg/Kg b.wt) caused non significant suppression of the hemolysing antibody titer in the treated dams ($G_2$ & $G_3$) compared with the control dams ($G_1$). On the contrary, aluminum chloride exposure of these dams caused significant suppression of the hemolysing antibody titer in their offsprings ($G_2$ & $G_3$) in relation to the offsprings from the control group ($G_1$).

***Effects on serum protein.*** Serum protein profile of control and treated dams and their offsprings was

**Table 1.** Hemolysin antibody titer and DTH reaction against SRBCs in control and treated dams and their offsprings

| Parameters | $Log_2$ hemagglutination titer | | | Diameter of skin reaction (mm) | | |
|---|---|---|---|---|---|---|
| Groups | $G_1$ | $G_2$ | $G_3$ | $G_1$ | $G_2$ | $G_3$ |
| Dams | 27.20 ± 7.71 | 22.40 ± 8.25 | 24.00 ± 8.40 | 0.341 ± 0.013 | 0.322 ± 0.019 | 0.336 ± 0.018 |
| Offsprings | 28.80 ± 6.73 | 13.60* ± 3.85 | 14.40* ± 3.36 | 0.263 ± 0.015 | 0.232 ± 0.009* | 0.241 ± 0.012* |

Values presents mean ± SD, N = 10.
*: Significant difference between control and treated groups at $p \leq 0.05$.
GI: Control group.
GII: Treated dams from 6th day of gestation - 21st day postpartum.
GIII: Treated dams from 15st day - 21st day postpartum.

**Table 2.** Protein profile of control and treated dams and their offsprings

| Parameter / Group | | Offsprings $G_1$ | Offsprings $G_2$ | Offsprings $G_3$ | Dams $G_1$ | Dams $G_2$ | Dams $G_3$ |
|---|---|---|---|---|---|---|---|
| Total protein g/dl | | 6.273 ± 1.030 | 6.288 ± 1.900 | 5.913 ± 1.830 | 8.322 ± 1.620 | 8.107 ± 2.060 | 7.786 ± 2.243 |
| Albumin g/dl | | 3.707 ± 0.900 | 3.949 ± 1.530 | 3.671 ± 1.450 | 5.470 ± 1.420 | 5.020 ± 1.520 | 5.101 ± 1.204 |
| Total globulins g/dl | | 2.569 ± 0.230 | 2.340* ± 0.130 | 2.243* ± 0.120 | 2.852 ± 0.933 | 3.088 ± 0.820 | 2.684 ± 0.594 |
| A/G ratio | | 1.443 ± 0.250 | 1.686* ± 0.310 | 1.637* ± 0.210 | 1.918 ± 0.523 | 1.625 ± 0.265 | 1.900 ± 0.489 |
| Globulins g/dl | α | 0.143 ± 0.040 | 0.173 ± 0.04 | 0.174 ± 0.060 | 0.325 ± 0.102 | 0.338 ± 0.123 | 0.306 ± 0.151 |
| | β | 1.190 ± 0.290 | 1.437 ± 0.400 | 1.370 ± 0.370 | 1.571 ± 0.570 | 1.501 ± 0.609 | 1.494 ± 0.439 |
| | γ | 1.236 ± 0.270 | 0.730* ± 0.030 | 0.699* ± 0.070 | 1.026 ± 0.470 | 0.980 ± 0.075 | 0.884 ± 0.224 |

Values presents mean ± SD, N = 10.
*: Significant difference between control and treated groups at $p \leq 0.05$.
GI: Control group.
GII: Treated dams from 6th day of gestation - 21st day postpartum.
GIII: Treated dams from 15st day - 21st day postpartum.

recorded in Table 2.

***Total proteins.*** Result of total protein showed non significant difference between the control ($G_1$) and treated dams ($G_2$ & $G_3$) and between the two treated groups themselves. This non significant difference was also recorded between the treated groups' offsprings and the corresponding controls.

***Serum protein fractions.*** Serum protein fractionation showed non significant difference among the different dams groups for albumin (A), total globulins (G), A/G ratio and globulin fractions. This non significant difference was also recorded for albumin, alpha and beta globulins of treated dam's offsprings compared with control. However, total globulins, A/G ratio and gamma globulin fractions showed significant differences in the treated dam's offsprings in comparison with the corresponding controls.

***Effects on histological structures of thymus, spleen and liver.*** There were no histopathological changes observed in thymus, spleen and liver of the control and treated dams.

Thymus of treated dam's offsprings (G2) showed lymphoid depletion of the medullary portion (Fig. 1). Their spleens showed lymphoid depletion in the white pulps while the red pulps were congested (Fig. 2). Moreover, the liver of this group showed dilation and congestion of its central vein with degenerative changes in the hepatocytes (Fig. 3).

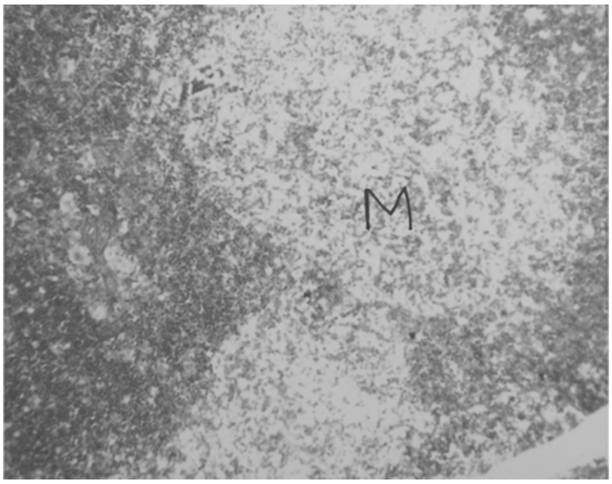

**Fig. 1.** Thymus of a rat fetus obtained from a mother treated orally with 345 mg Al chloride/Kg b. wt. on the 6th day of gestation till weaning (21st day postpartum) showing depletion in the lymphoid cells of medullary portion (M). (H & E stain, × 40).

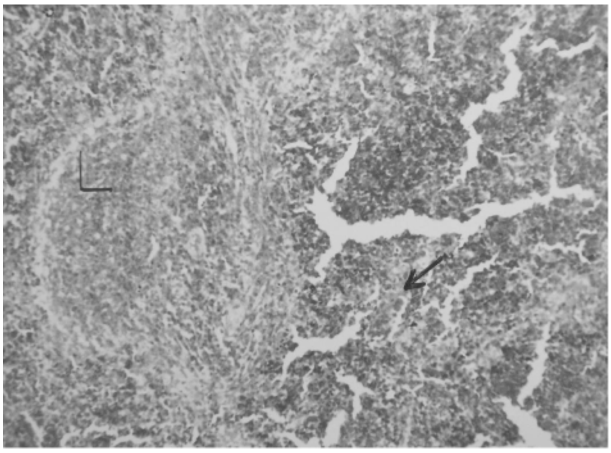

**Fig. 2.** Spleen of a rat fetus obtained from a mother treated orally with 345 mg Al chloride/Kg b. wt. on the 6th day of gestation till weaning (21st day postpartum) showing depletion in lymphoid cells in white pulps (L) and congestion in red pulps (arrow). (H & E stain, × 40).

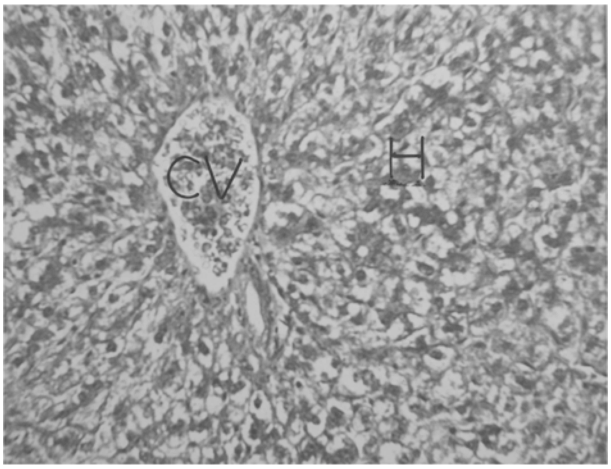

**Fig. 3.** Liver of a rat fetus obtained from a mother treated orally with 345 mg Al chloride/Kg b. wt. on the $6^{th}$ day of gestation till weaning ($21^{st}$ day postpartum) showing dilatation and congestion in the central vein (CV) with degeneration in the hepatocytes (H). (H & E stain, × 64).

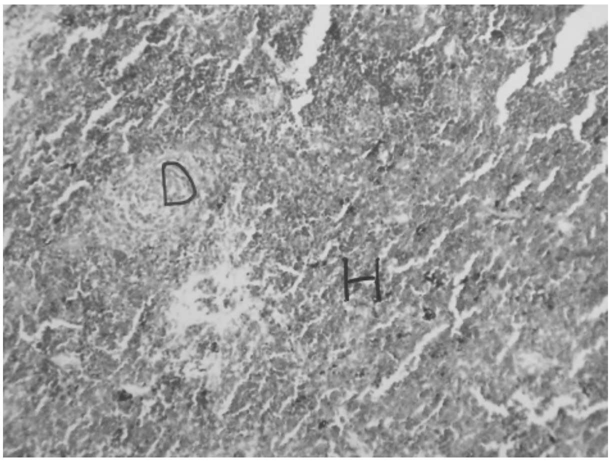

**Fig. 5.** Spleen of a rat fetus obtained from a mother treated orally with 345 mg Al chloride/Kg b. wt. on $15^{th}$ day of gestation to the end of weaning showing lymphoid depletion in white pulps (D) with congestion and hemosidrosis in red pulps (H) (H & E stain, × 40).

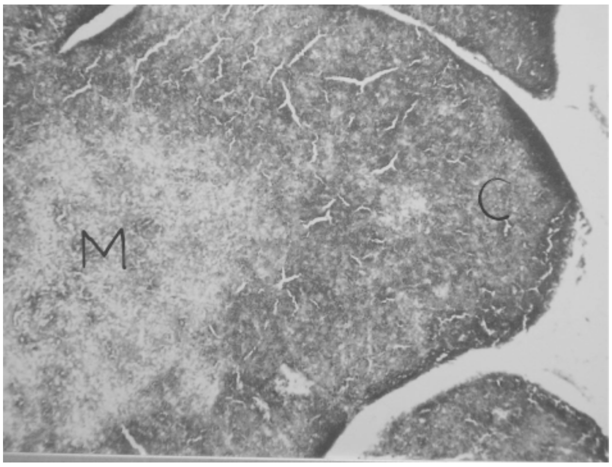

**Fig. 4.** Thymus of a rat fetus obtained from a mother treated orally with 345 mg Al chloride/Kg b. wt. on $15^{th}$ day of gestation to the end of weaning showing lymphoid depletion in both cortex (C) and medulla (M). (H & E stain, × 25.5).

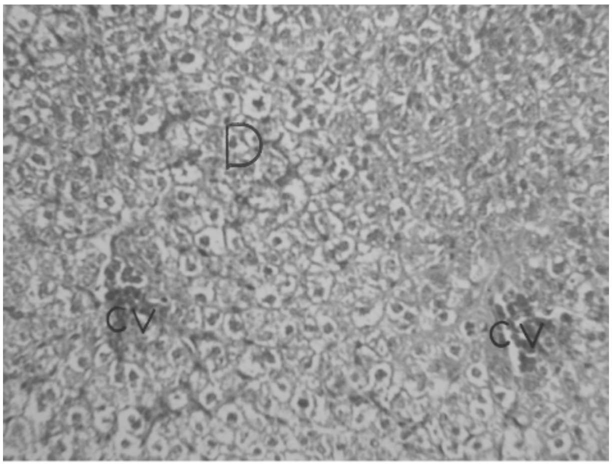

**Fig. 6.** Liver of a rat fetus obtained from a mother treated orally with 345 mg Al chloride/Kg b. wt. on $15^{th}$ day of gestation to the end of weaning showing degeneration in the hepatocytes (D) with congestion in central vein (CV). (H & E stain, × 40).

Offsprings of (G3) treated dams showed lymphoid depletion in both thymus cortex and medulla (Fig. 4). Also, the red pulps of their spleens showed congestion with hemosiderosis while the white pulps had lymphoid depletion (Fig. 5). Their livers showed congestion in central vein with degeneration of the surrounding hepatocytes (Fig. 6).

## DISCUSSION

It is established that certain metals and metal-based compounds are inherently toxic, and their presence in occupational and environmental settings raises appropriate questions concerning human exposure. They can alter the immune response of laboratory animals and probably humans as well (Hostek *et al.*, 1993). In some instances, the immune system appears to be exquisitely sensitive to these agents compared to other toxicological parameters (Golub *et al.*, 1993). Although the majority of data accumulated to date pertains to effects in small laboratory rodents, there is little reason to believe that similar quantifiable effects do not occur in domestic and food-producing animals due to basic functional similarities of the immune system of mammals in general (Exon, 1984). Here we examined the effect of Al chloride at the teratogenic dose level (345 mg/Kg b. wt.) (Sharma and Mishra, 2006) on the immune system of the aluminum exposed dams and their offsprings. It was evident that there was a non-significant difference in DTH reaction (cell-mediated immune response), hemolysin antibody titer (humoral immune response), serum total protein and protein fractions between aluminum-exposed dams and those of control dams, confirming the absence of any immunosuppressant effect of Al in the exposed dams. This was sustained by the normal histological findings in thymus, spleen and liver of these treated dams. On the other hand, aluminum chloride exposure of these dams caused significant inhibition of the cell mediated and humoral immune responses, serum total globulins, A/G ratio and gamma globulin fraction in their offsprings in relation to the offsprings from the control group. This was confirmed by the recorded histopathological alterations in thymus, spleen and liver.

Our result concerning the absence of any hepatic histological alteration and the insignificant decrease in serum total protein of treated dams are similar to those recorded by Fontana *et al.* (1991) in rabbits following long-term exposure to tris (maltolate) aluminum (III). On the other side, Cherroret *et al.* (1995) confirmed the suppressive effects of aluminum chloride on plasma total protein concentration in normal and uremic adult male rats.

The observed immunosuppressive effects in aluminum-exposed offsprings are in agreement with Davenport *et al.* (1989); Garrett (1989); OEHHA (1999) and ATSDR (2006). In addition, Nordal *et al.* (1989) and Msarcpondes *et al.* (1996) attributed the observed rejection episode in kidney Allograft recipients to Al accumulation in bone, suggesting its immunosuppressive effect. The immunosuppressive effect of Al is confirmed by the recorded histopathological alterations in the thymus gland and spleen of Al chloride-treated dams' offspring. Similar pathological alterations were observed in spleen and liver of rats (Gomez *et al.*, 1986; Stein *et al.*, 1987) and rabbits (Favarato and Zatta, 1993) following Al exposure. Nikolova *et al.* (1994) recorded different functional and morphological changes in the liver of white rats treated with aluminum. They reported that the histochemical data argued for disorders of protein metabolism.

In coincidence with the observed effects of Al on cellular immune response, it has been recorded that asthma and bronchial hyperactivity usually occur on exposure to aluminum salts in air at concentrations below 1 mg/m$^3$ (Nordic expert group, 1993). Lopez *et al.* (1994) concluded that delayed sensitivity to aluminum appears to be implicated in the pathogenesis of persistent nodular reactions in humans during hyposensitization therapy with aluminum-precipitated antigen solutions. The contact sensitivity to aluminum was confirmed by Helgesen and Austad (1997) and Peters *et al.* (1998). Bergfors *et al.* (2005) observed that intensely itching subcutaneous nodules, lasting for many years and hypersensitivity to aluminum sometimes occur after the use of several Al adsorbed vaccines in infants. Bytautiene *et al.* (2005) showed that maternal exposure to aluminum hydroxide during pregnancy altered the immune responses and pulmonary function in the offsprings later in life, with mechanical and pathological findings indicative of type I hypersensitivity responses. Fetal programming occurring in utero was attributed to be responsible for the manifestation of type I hypersensitivity reactions, such as asthma and hives, later in life. Ward *et al.* (2006) mentioned that short term (< 10 days) exposure to episodic pulses of aqueous Al increases the risk of infection in the crayfish by impairing the ability of haemocytes to recognize and/or remove bacteria from the circulation. These literatures could approve the Al- induced suppressive effect on cell mediated- immune response in our findings.

Humoral immunity is the aspect of immunity that is mediated by secreted antibodies, produced in the cells of the B lymphocyte lineage (B-cell). Secreted antibodies bind to antigens on the surfaces of invading microbes (such as viruses or bacteria), which flags them for destruction (Pier *et al.*, 2004). Eginchibaerva (1988) concluded that mother's milk antibodies induce immunosuppressive effect on the parameters of the humoral immune response of the progeny and on the degree of their sensitization. In offspring derived from mothers immunized by a suitable amount of T-dependent antigen, clear-cut suppression of development of specific plaque forming cells (PFC) in spleen was observed over a significant period after delivery (Watanabe *et al.*, 1984).

The delayed type hypersensitivity reaction, a parameter of cell mediated immunity, and the humoral immune response against SRBC, which needs the co-operation of T-helper cells and B-cells, were distinctly depressed by aluminum chloride in rat offspring. Therefore, aluminum chloride exposure of dams at the tested dose level may suppress the offspring-immune response by affecting both B and T cells function. This suggestion is confirmed by the observed lymphoid depletion in thymus and spleen. Seinen and Willems (1976) reported that the antibody production against SRBCs is reduced by the impairment of T-helper cell function and/or impairment of T-effector cells (another subpopulation of T-lymphocytes). The recorded suppression of cell mediated immune response as evidenced by the significant reduction in DTH reaction to SRBCs could be attributed to an effect on T-cells (T-effector cells) subpopulation (Danneberg, 1991). Golub *et al.* (1993) recorded a deficit in immune effector cell function after long term in vivo aluminum exposure of mice.

From these results, it can be concluded that aluminum chloride exposure of dams resulted in immune suppression in their offsprings, probably by an effect on T and/or B-cells. This effect was more pronounced after treatment with the tested chemical during the developmental phase of the lymphoid system. Additional support for the severe consequence of early exposure comes from the observation that aluminum chloride intubation of dams at 15$^{th}$ day gestation caused mild alteration of immune parameters of their offsprings than in those of treated dams at the 6$^{th}$ day of gestation, although this difference was non-significant. This observation confirmed those of Lauricella *et al.* (2001) who mentioned that Al effects on the immune system might depend on the dose, route of administration and length of exposure, as well as on the cell population assayed.

## ACKNOWLEDGMENT

The authors are greatly indebted to Dr. A. Bakeer, Prof. of Pathology, Faculty of Veterinary Medicine, Cairo University for her help on the histopathological examination.

## References

[CR1] A. T. S. D. R. (2006). Toxicological Profile for Aluminum (Update). Draft for public comment. Agency for Toxic Substances and Disease Registry, Public Health Service.

[CR2] Bancroft JD, Stevens A, Turner DR (1996). Theory and practice of histological techniques.

[CR3] Barr RJ, Alpern KS, Jay S (1993). Histocytic reaction associated with topical aluminum chloride (Drysol reaction). J. Dermatol. Surg. Oncol..

[CR4] Bergfors E, Björkelund C, Trollfors B (2005). Nineteen cases of persistent pruritic nodules and contact allergy to aluminium after injection of commonly used aluminum-adsorbed vaccines. Eur. J. Pediatr.

[CR5] Bomford R (1980). The comparative selectivity of adjuvants for humoral and cell-mediated immunity. Clin. Exp. Immunol..

[CR6] Bytautiene E, Long M, Orise P, Hankins G, Anderson G, Saade G (2005). Fetal programming of type I hypersensitivity reaction: The roles of leukotriene D4, prostaglandin D2 and thromboxane A2. Am. J. Obstet. Gynecol..

[CR7] Carson BL (2000). Aluminum compounds: Review of toxicological literature. Abridged final report, National Institute of Environmental Health Sciences.

[CR8] Cherroret G, Capolaghi B, Hutin MF, Burnel D, Desor D, Lehr PR (1995). Effects of postnatal aluminum exposure on biological parameters in the rat plasma. Toxicol. Lett..

[CR9] Comoy EE, Capron A, Thyphronitis G (1997). *In vivo* induction of types 1 and 2 immune responses against protein antigens. Int. Immunol..

[CR10] Danneberg AM (1991). Delayed-type hypersensitivity and cell mediated immunity in the pathogenesis of tuberculosis. Immunol. Today.

[CR11] Davenport A, Davison AM, Will EJ, Toothill C, Newton KE, Giles GR (1989). Aluminum accumulation and immunosuppression. BMJ.

[CR12] Dean JH, Cornacoff JB, Rosenthal GJ, Luster MI (1989). Immune system, evaluation of injury. Principles and Methods of Toxicology.

[CR13] Domingo JL (1995). Reproductive and developmental toxicity of aluminum: a review. Neurotoxicol. Teratol..

[CR14] Eginchibaerva RG (1988). Modulation of the humoral immune response in rats by antibodies from maternal milk. Vopr. Pitan.

[CR15] Exon JH (1984). The immunotoxicity of selected environmental chemicals, pesticides and heavy metals. Prog. Clin. Biol. Res..

[CR16] Favarato M, Zatta PF (1993). Differential aluminum lactate toxicity in rabbits using either aqueous solution or liposomal suspensions. Toxicol. Lett..

[CR17] Fontana L, Perazzlo M, Stella M, Tapparo A, Corain B, Favarato M, Zatta P (1991). A long-term toxicological investigation on the effect of tris (maltolate) aluminum (III) in rabbits. Biol. Trace. Elern. Res..

[CR18] Ganrot PO (1986). Metabolism and possible health effects of aluminum. Environ. Health Perspect..

[CR19] Garrett P (1989). Aluminum accumulation and immunosuppression. BMJ.

[CR20] Golub MS, Takeucchi PT, Gershwin ME, Yoshida SH (1993). Influence of dietary aluminum on cytokine production by mitogen-stimulated spleen cells Swiss Webster mice. Immunopharmacol. Immunotoxicol..

[CR21] Gomez M, Domingo JL, Llobet JM, Thomas JM, Corbella J (1986). Short-term oral toxicity study of aluminum in rats. Arch. Pharmacol. Toxicol..

[CR22] Helgesen AL, Austad J (1997). Contact urticaria from aluminum and nickle in the same patient. Contact Dermatitis.

[CR23] Hostek JJ, Hinz RS, Loreence CR, Price M, Guy RH (1993). Metals and skin. Cht. Rev. Toxicol..

[CR24] Keyser JW, Watkins GL (1972). Estimation of serum proteins by electrophoresis on cellulose acetate. Clin. Chem.

[CR25] King RJ, Wooton LDP (1959). Microanalysis. Medical Biochemistry.

[CR26] Lauricella AM, Garbossa G, Nesse A (2001). Dissimilar behavior of lymph cells in response to the action of aluminium. *In vitro* and *in vivo* studies. Int. Immunophar-macol..

[CR27] Lione A (1985). Aluminum toxicology and the aluminum-containing medications. Pharm. Then.

[CR28] Lopez S, Pelaez A, Navarro LA, Montesinos E, Morales C, Carda C (1994). Aluminum allergy in patients hyposensitized with aluminum-precipitated antigen extracts. Contact Dermatitis.

[CR29] May JC, Rains TC, Maienthal FJ, Biddle GN, Progar JJ (1986). A survey of the concentrations of eleven Metals in vaccines, allergenic extracts, toxoids, blood, blood derivatives and other biological products. J. Biol. Standardization.

[CR30] Misawa T, Shigeta S (1992). Behavioral effects of repeated aluminum administration in the rat. Tokai J. Exp. Clin. Med..

[CR31] Monis B, Valentich MA (1993). Promoting effects of mancozeb on pancreas of nitrosomethylurea treated rats. Carcinogenesis.

[CR32] Msarcpondes M, Jorgetti V, Futata E, Orii N, Azevedo L S, Tzanno-Martins C, Duarte AJ (1996). The role of experimental chronic renal failure and intoxication in cellular immune response. Nephrol. Dial. Transplant..

[CR33] Nikolova P, Softova E, Kavaldzhieva B, Boiadzhieva S (1994). The functional and morphological changes in the liver and kidneys of white rats treated with aluminum. Eksp. Med. Morfol..

[CR34] Nordal KP, Dahl E, Albrechtsen D, Halse J, Leivestad T, Tretli S, Flatmark A (1989). Aluminum accumulation and immunosuppressive effect in recipients of kidney transplants. BMJ.

[CR35] Nordic expert group, S.B. (1993). Aluminum. Arbete Och. Ha..

[CR36] OEHHA Office of Environmental Health Hazard Assessment (1999). Draft for review only public health goal for aluminum in drinking water.

[CR37] Peters T, Hani N, Kirchberg K, Gold H, Hunzelmann N, Scharffetter-Kochanek K (1998). Occupational contact sensitivity to aluminum in a machine construction plant worker. Contact Dermatitis.

[CR38] Pier GB, Lyczak JB, Wetzler LM (2004). Immunology, Infection, and Immunity.

[CR39] Schafer SG, Dawes RIF, Elsenhans B, Forth W, Schumann K (1999). Metals “in” Toxicology edited by Hans Marquardt, Siegfried G. Schafer, Roger McClellan & Frank Welsch.

[CR40] Seinen W, Willems MI (1976). Toxicity of organotin compounds. I. Atrophy of thymus and thymus dependent lymphoid tissue in rats fed di-*N*-octyltin dichloride. Toxicol. Appl. Pharmacol..

[CR41] Seinen W, Vos JG, Kricken RV, Penninks A, Brands R, Hooykaas H (1977). Toxicity of organotin compounds. III. Suppression of thymus - dependent immunity in rats by di-n-butyltindichloride and di-n-octytindichloride. Toxicol Appl. Pharmacol..

[CR42] Sharma P, Mishra KP (2006). Aluminum-induced maternal and developmental toxicity and oxidative stress in rat brain: response to combined administration of Tiron and glutathione. Reprod. Toxicol..

[CR43] Stein Günter, Laske Volker, Müller Andreas, Bräunlich Helmut, Linß Werner, Fleck Christian (1987). Aluminium induced damage of the lysosomes in the liver, spleen and kidneys of rats. Journal of Applied Toxicology.

[CR44] Synzynys BI, Sharetskii AN, Kharlamova OV (2004). Immunotoxicity of aluminum chloride. Gig. Sanit..

[CR45] Tsundoa M, Sharma RP (1999). Modulation of tumor necrosis factor Alpha expression in mouse brain after exposure to aluminum in drinking water. Arch. Toxicol.

[CR46] Ward RJ, McCrohan CR, White KN (2006). Influence of aqueous aluminum on the immune system of the freshwater crayfish Pacifasticus leniusculus. Aquat. Toxicol..

[CR47] Watanabe Y, Shimizu S, Yamaguchi N (1984). Effect of maternal antigenic stimulation on the active immune response of their offspring: Relationship between the immune reactivity of mother mice and the induction of suppression in their young. Scand. J. Immunol..

[CR48] WHO World Health Organization (1997). Aluminum, international programs on chemical safety environmental health criteria.

[CR49] Zaman K, Zaman A, Batcabe J (1993). Hematological effects of aluminum on living organisms. Comp. Biochem. Physiol. C.

